# Clinical factors associated with unipolar mania: A systematic review and meta-analysis

**DOI:** 10.1192/j.eurpsy.2023.1075

**Published:** 2023-07-19

**Authors:** F. Bartoli, C. Nasti, D. Palpella, S. Piacenti, M. E. Di Lella, S. Mauro, L. Prestifilippo, C. Crocamo, G. Carrà

**Affiliations:** 1 Department of Medicine and Surgery, Università degli Studi di Milano - Bicocca, Milan, Italy; 2 Division of Psychiatry, University College London, London, United Kingdom

## Abstract

**Introduction:**

The existence of a clinical entity on the spectrum of mood disorders characterized by the occurrence of manic episodes without major depressive episodes (Unipolar Mania, UM) is largely debated. Although not classified nosologically, the studies exploring this topic have suggested that UM might differ from bipolar disorder with a manic-depressive course (md-BD), in terms of several clinical characteristics. Individuals with UM might represent a subpopulation with specific clinical profiles and unmet care needs, requiring personalized treatments, as compared with those suffering from md-BD.

**Objectives:**

To identify clinical factors associated with UM, as compared with md-BD.

**Methods:**

We performed a systematic review and meta-analysis of observational studies according to the MOOSE guidelines. We searched for articles indexed up to July 2022 in the main electronic databases. We conducted random-effects meta-analyses of the association between UM and relevant correlates, using odds ratio for categorical variables and standardized mean difference for continuous variables.

**Results:**

Based on data from 21 studies meeting the eligibility criteria, we found that individuals with UM, as compared with md-BD, were more likely to be males (p = 0.007) and to have an earlier age at onset (p = 0.020). Moreover, UM was significantly associated with a higher number of hospitalizations (p < 0.001), the occurrence of psychotic features (p < 0.001), as well as hyperthymic temperament (p = 0.012). Finally, subjects with UM were less likely to report a family history of depression (p = 0.006) and a personal history of suicide attempts (p < 0.001).

**Conclusions:**

Our work supports the hypothesis that UM might represent a distinctive diagnostic construct, with peculiar clinical correlates. Additional research is needed to better differentiate UM in the context of affective disorders.

**Disclosure of Interest:**

None Declared

